# Predicting the Significance of Necessity

**DOI:** 10.3389/fpsyg.2019.00283

**Published:** 2019-02-13

**Authors:** Kimmo Sorjonen, Bo Melin

**Affiliations:** Karolinska Institute, Solna, Sweden

**Keywords:** necessary condition analysis, permutation, power, significance testing, simulation, sufficiency

## Abstract

With Necessary Condition Analysis (NCA), a necessity effect is estimated by calculating the amount of empty space in the upper-left corner in a plot with a predictor *X* and an outcome *Y*, and recently a method for testing the statistical significance of the necessity effect through permutation has been proposed. In the present simulation study, this method was found to give significant results already with a very weak true population necessity effect, i.e., exhibit high power, unless the sample size is very small. However, in some situations the significance of the necessity effect tends to increase with increased degree of sufficiency, which is paradoxical for a method whose objective is to find necessary but not sufficient conditions.

## Introduction

**FIGURE 1:**
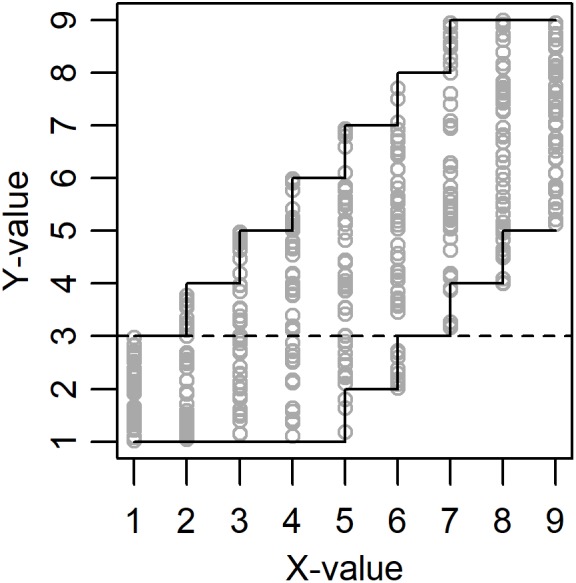
Example of data (*N* = 450) drawn from a population with true necessity effect = 0.328 (amount of empty space in the upper-left corner) and true sufficiency effect = 0.156 (amount of empty space in the lower-right corner). See the discussion for the meaning of the dashed line.

Necessary Condition Analysis (NCA) has been developed to help researchers identify necessary but not sufficient conditions for an outcome *Y* of interest (Dul, 2016). The calculated necessity effect corresponds to the amount (percentage) of empty space in the upper-left corner when plotting outcome *Y* against a predictor *X* (Figure 1). A large empty space in the upper-left corner is taken to indicate that a certain minimum level of *X* is necessary for a high level of *Y*. Different functions can be applied to so called ceiling points (data points with a *Y*-value that is higher than the *Y*-value for all data points with a lower *X*-value), but in the present study we will stick to a step-function named Ceiling Envelopment-Free Disposal Hull (CE-FDH), which has been proposed as the default technique for NCA. CE-FDH values below 0.1 has been described as small, values between 0.1 and 0.3 as medium, values between 0.3 and 0.5 as large, and values above 0.5 as very large necessity effects (Dul, 2016). In Figure 1 the size of the empty space in the upper-left corner equals 6 + 5 + 4 + 3 + 2 + 1 = 21 and the size of the full plot space equals (9-1) × (9-1) = 64. Hence, the necessity effect equals 21/64 = 0.328.

Studies using NCA have claimed that a certain level of intelligence is necessary for creativity (Karwowski et al., 2016, 2017; Shi et al., 2017), that a certain degree of workplace spirituality is necessary for high levels of employee commitment, job satisfaction, and work-life balance satisfaction (Garg, 2017), that contracts with at least medium levels of contractual detail as well as the highest levels of trust are necessary for buyer-supplier relationships that have high levels of innovation (Van der Valk et al., 2016), that a certain minimum level of safety consciousness is required to achieve top productivity results among long-haul truck drivers (de Vries et al., 2017), and that a certain level of metacognition is necessary for the emergence of motivation in people with schizophrenia spectrum disorders (Luther et al., 2017).

Qualitative Comparative Analysis (QCA; Ragin, 1987) is another method that has been used to detect necessary conditions, and according to Thiem (2018) QCA is superior to NCA, however, see the response by Dul et al. (2018b). We have shown previously that with a negatively skewed predictor *X* and a positively skewed outcome *Y*, NCA can give large necessity effects even if *X* and *Y* are unrelated. The reason is that this combination of skewness results in a low probability to get data points in the upper-left corner in an *X*–*Y*-plot (Sorjonen et al., 2017). Recently, Dul et al. (2018a) have proposed that *p*-values for necessity effects can be calculated with so called permutation tests in order to guard against type 1-errors. In a permutation test data, in this case the *Y*-variable (but not the *X*-variable), is reshuffled randomly (i.e., random sampling without replacement) a large number of times and the parameter of interest, in this case the necessity effect, is calculated after each reshuffle. The calculated *p*-value corresponds to the proportion of parameter-values that are equal to or stronger than the observed parameter-value. In the example in Table 1 the calculated *p*-value for CE-FDH would be 4/10 = 0.4.

**Table 1:** An example with ten permutations of the observed *Y*-values.

*X*	*Y*-values										
	Obs.	P1	P2	P3	P4	P5	P6	P7	P8	P9	P10
1	3	2	4	3	4	10	5	7	3	10	5
1	7	7	1	4	3	4	2	4	3	7	7
1	4	4	4	4	5	4	4	4	7	5	3
1	1	10	10	3	3	1	10	5	5	7	10
2	4	1	7	7	1	2	1	10	4	4	4
2	3	4	3	1	7	5	3	3	2	2	4
2	1	1	2	10	4	3	4	7	1	1	3
2	10	1	1	2	1	3	7	3	7	1	1
3	1	5	5	7	10	1	3	1	1	4	1
3	7	3	3	5	2	7	1	1	4	3	1
3	2	7	7	1	1	1	7	2	10	3	2
3	5	3	1	1	7	7	1	1	1	1	7
CE-FDH	0.167	0.000	0.000	**0.333**	**0.444**	0.000	0.000	**0.167**	**0.333**	0.000	0.000


*As four permuted values are at least as strong as the evaluated CE-FDH = 0.167 (bold), the p-value would be 4/10 = 0.4.*

The objective of the present study was to evaluate if and how calculated *p*-values for necessity effects can be predicted from true population necessity effect (i.e., amount of empty space in the upper-left corner in an *X*–*Y*-plot), true population sufficiency effect, and sample size. This gives indications of the power of the method as well as risk for type 1-errors. Degree of sufficiency was operationalized as amount of empty space in the lower-right corner in an *X*–*Y*-plot, something that indicates that a certain minimum level of *X* precludes a low value on *Y*.

## Method

In the present simulations (script and data available from https://osf.io/kefxd/), between 18 and 495 virtual subjects were assigned an *X*-value between 1 (min) and 9 (max), same *N* for all *X*-values (i.e., a discrete uniform distribution). Then all virtual subjects were assigned a *Y*-value from a random continuous uniform distribution. As a mock example, the discrete *X*-variable from 1 to 9 could be self-rated depression on a single Likert-type scale while the continuous *Y*-variable could be suicidal ideation measured with a multi-item questionnaire resulting in a continuous score, and the research question could be if a certain minimum level of depression is necessary for a high degree of suicidal ideation.

True population necessity effect was defined by the upper limit for *Y* when *X* = 1. The upper limit for *Y* when *X* = 2 was always set to be the upper limit when *X* = 1 + 1 or 9, whichever was lowest. The upper limit for *Y* when *X* = 3 was always set to be the upper limit when *X* = 1 + 2 or 9, whichever was lowest, etc. So, for example, the upper limits for *Y* when *X* = 1 to 9 could be 3, 4, 5, 6, 7, 8, 9, 9, and 9, respectively, and this gave a true population necessity effect = 0.328 (Figure 1). True population sufficiency effect was defined by the lower limit for *Y* when *X* = 9. The lower limit for *Y* when *X* = 8 was always set to be the lower limit when *X* = 9-1 or 1, whichever was highest. The lower limit for *Y* when *X* = 7 was always set to be the lower limit when *X* = 9-2 or 1, whichever was highest, etc. So, for example, the lower limits for *Y* when *X* = 1 to 9 could be 1, 1, 1, 1, 1, 2, 3, 4, and 5, respectively, and this gave a true population sufficiency effect = 0.156 (Figure 1). The true degree of necessity and sufficiency for all 81 combinations of upper and lower limits is presented in Table 2.

**Table 2:** The 81 combinations of true population necessity (N) and sufficiency (S) effects used in the present study.

	Upper limit for *Y* when *X* = 1									
		1	2	3	4	5	6	7	8	9
**Lower limit for *Y* when *X* = 9**	1	N = 0.563	N = 0.438	N = 0.328	N = 0.234	N = 0.156	N = 0.094	N = 0.047	N = 0.016	N = 0.000
		S = 0.000	S = 0.000	S = 0.000	S = 0.000	S = 0.000	S = 0.000	S = 0.000	S = 0.000	S = 0.000
	2	N = 0.563	N = 0.438	N = 0.328	N = 0.234	N = 0.156	N = 0.094	N = 0.047	N = 0.016	N = 0.000
		S = 0.016	S = 0.016	S = 0.016	S = 0.016	S = 0.016	S = 0.016	S = 0.016	S = 0.016	S = 0.016
	3	N = 0.563	N = 0.438	N = 0.328	N = 0.234	N = 0.156	N = 0.094	N = 0.047	N = 0.016	N = 0.000
		S = 0.047	S = 0.047	S = 0.047	S = 0.047	S = 0.047	S = 0.047	S = 0.047	S = 0.047	S = 0.047
	4	N = 0.563	N = 0.438	N = 0.328	N = 0.234	N = 0.156	N = 0.094	N = 0.047	N = 0.016	N = 0.000
		S = 0.094	S = 0.094	S = 0.094	S = 0.094	S = 0.094	S = 0.094	S = 0.094	S = 0.094	S = 0.094
	5	N = 0.563	N = 0.438	N = 0.328	N = 0.234	N = 0.156	N = 0.094	N = 0.047	N = 0.016	N = 0.000
		S = 0.156	S = 0.156	S = 0.156	S = 0.156	S = 0.156	S = 0.156	S = 0.156	S = 0.156	S = 0.156
	6	N = 0.563	N = 0.438	N = 0.328	N = 0.234	N = 0.156	N = 0.094	N = 0.047	N = 0.016	N = 0.000
		S = 0.234	S = 0.234	S = 0.234	S = 0.234	S = 0.234	S = 0.234	S = 0.234	S = 0.234	S = 0.234
	7	N = 0.563	N = 0.438	N = 0.328	N = 0.234	N = 0.156	N = 0.094	N = 0.047	N = 0.016	N = 0.000
		S = 0.328	S = 0.328	S = 0.328	S = 0.328	S = 0.328	S = 0.328	S = 0.328	S = 0.328	S = 0.328
	8	N = 0.563	N = 0.438	N = 0.328	N = 0.234	N = 0.156	N = 0.094	N = 0.047	N = 0.016	N = 0.000
		S = 0.438	S = 0.438	S = 0.438	S = 0.438	S = 0.438	S = 0.438	S = 0.438	S = 0.438	S = 0.438
	9	N = 0.563	N = 0.438	N = 0.328	N = 0.234	N = 0.156	N = 0.094	N = 0.047	N = 0.016	N = 0.000
		S = 0.563	S = 0.563	S = 0.563	S = 0.563	S = 0.563	S = 0.563	S = 0.563	S = 0.563	S = 0.563


*Necessity effect is defined by the upper limit for Y when X = 1 and sufficiency effect by the lower limit for Y when X = 9.*

In a number of simulations (see result section) the *p*-value of the observed necessity effect was calculated through 1000 permutations. Using logistic regression, the odds for a significant necessity effect (*p* < 0.05) was predicted from the true population necessity effect, square root of sample size, and/or true population sufficiency effect. The predicted odds was transformed to predicted probability to get a significant result, i.e., predicted power. Simulations and analyzes were carried out with R 3.5.0 statistical software (R Core Team, 2018), using the NCA 3.0 package (Dul, 2018).

## Results

### Without Sufficiency

**FIGURE 2:**
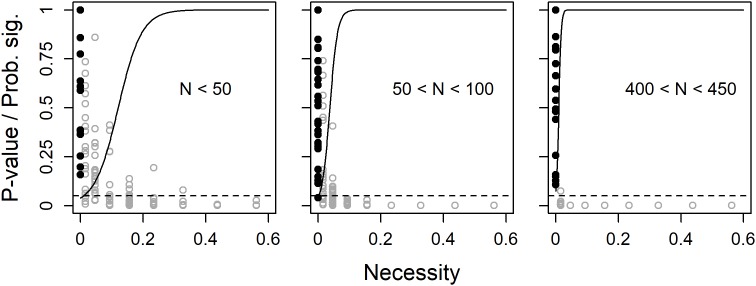
Observed *p*-values (dots) and predicted probability for a significant (*p* < 0.05) necessity effect (solid line) as functions of true population necessity effect, separately for three ranges of sample size. The filled black dots are observed *p*-values when true degree of necessity = 0 and the dashed line shows the limit for *p* < 0.05.

In a first set of 2000 simulations, the true population sufficiency effect was fixed at zero (i.e., no empty space in the lower-right corner). Unless with a very small sample size, the probability for a significant necessity effect, i.e., power, was found to quickly approach unity with an increased true population necessity effect. With a true population necessity effect = 0, the analysis did not give significant results, i.e., type 1-errors, more often than expected (5%) (Figure 2).

### With Sufficiency

**FIGURE 3:**
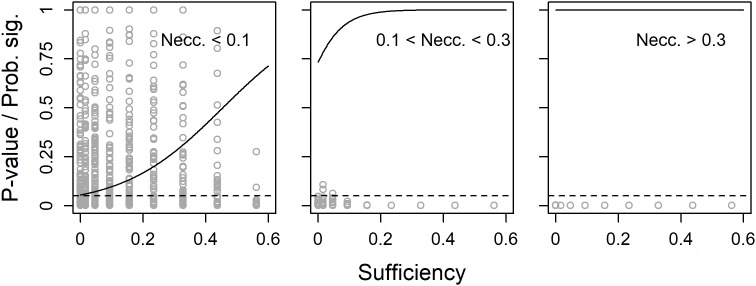
Observed *p*-values (dots) and predicted probability for a significant (*p* < 0.05) necessity effect (solid line) as functions of true population sufficiency effect, separately for three ranges of true population necessity effect. *N* = 45 and the dashed line shows the limit for *p* < 0.05.

In a second set of 2000 simulations, also the true population sufficiency effect was allowed to vary. However, as the analyses above indicated low degree of variance in *p*-values with large, or even not so large, samples, sample size was fixed at a low *N* = 45. Unless the true population necessity effect was quite large, the probability to get a significant observed necessity effect increased with increased true population sufficiency effect (Figure 3).

### Without Necessity

**FIGURE 4:**
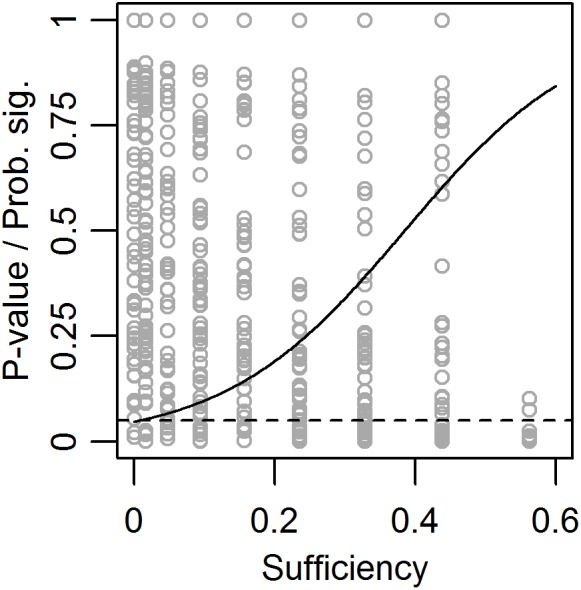
Observed *p*-values (dots) and predicted probability for a significant (*p* < 0.05) necessity effect (solid line) as functions of true population sufficiency effect when the true population necessity effect = 0. The dashed line shows the limit for *p* < 0.05.

The leftmost plot in Figure 3 indicates that the risk for type 1-error when calculating the significance of necessity effects might be influenced by the true population sufficiency effect. Therefore, in a third set of 500 simulations, the true population necessity effect was fixed at zero, while the true population sufficiency effect and sample size were allowed to vary. While sample size had no effect on the probability to get a significant observed necessity effect, i.e., the risk for type 1-error, this risk increased with increased true population sufficiency effect (Figure 4).

## Discussion

The present simulation indicates that unless they have a very small sample, users of NCA are more or less guaranteed a significant result already with a very small true necessity effect between *X* and *Y* in the population in question. Of course, this apparent high power of NCA could be seen as a positive characteristic. However, one might also become a bit worried by the ease with which people wanting to claim that *X* is a necessary condition for *Y* can overcome the obstacle of significance. Dul et al. (2018a) recommend that three criteria should be fulfilled before concluding that *X* is necessary for *Y*: (i) theoretical justification, (ii) observed necessity effect >0, and (iii) small *p*-value (e.g., *p* < 0.05). However, as criterion (iii) is sufficient for criterion (ii) (the *p*-value cannot be small if the effect equals zero), and as a rich literature on hindsight bias (see, for example, Guilbault et al., 2004) indicates that we humans are good at justifying observed associations after the fact, we would be left with the apparently quite indulgent criterion (iii) According to Mayo (1996), scientific hypotheses should be subjected to severe testing, “with an overwhelmingly good chance of revealing the presence of a specific error, if it exists – but not otherwise” (p. 7), and the question is if analyses with NCA, with or without significance testing, could be said to follow Mayo’s recommendation. For example, the present study indicates that the significance of an analysis with NCA will, in some situations, tend to increase with increased degree of sufficiency, which is paradoxical for a method whose objective is to identify necessary but not sufficient conditions for an outcome *Y*. As insufficiency is a necessary condition for “necessary but not sufficient” the significance of results from NCA should, on the contrary, decrease with increased degree of sufficiency.

A probable reason why degree of sufficiency has a positive effect on the calculated significance of the necessity effect is illustrated in Figure 1. In the permutation data points can move horizontally but not vertically, with the consequence that only data points above the dashed line can end up in the empty space in the upper-left corner and thereby decrease the calculated necessity effect and *p*-value. With high degree of sufficiency, i.e., a large empty space in the lower-right corner, a bigger proportion of data points can be found above the dashed line and this results in a lower calculated *p*-value. We recommend the developers of NCA to consider if this is a desirable consequence of how the significance of the necessity effect is calculated.

In the present simulation, subjects were uniformly distributed on the discrete *X*-variable and also uniformly distributed on the continuous *Y*-variable for all levels of *X*. It is unclear if, and how, the result would be affected by using some other distribution, for example truncated normal, exGaussian (common for response times), log-normal (income, city sizes), Poisson (number of goals in soccer games), or Weibull (manufacturing times) distributions.

All this said, maybe we should end on a more positive note: (i) Mayo’s recommendation is probably not met by a majority of all conclusions based on statistical significance testing (see, for example, Szucs and Ioannidis, 2017), so this critique is not specific for NCA; (ii) Without any true population sufficiency effect, NCA did not seem to result in more type 1-errors than expected, i.e., 5%; (iii) Although it’s extremeness in the present case makes us a bit suspicious and desiring more scrutiny, high power is, of course, desirable for a test of statistical significance.

## Author Contributions

KS carried out the simulations and analyses, and wrote an initial draft. KS and BM conceived of the presented idea discussed the results and contributed to the final manuscript. Both authors have approved the final version of the manuscript.

## Conflict of Interest Statement

The authors declare that the research was conducted in the absence of any commercial or financial relationships that could be construed as a potential conflict of interest.
